# Comparative Study of Bacteriophage Pharmacokinetics by Different Enteral Administration Routes

**DOI:** 10.3390/mps9030089

**Published:** 2026-06-03

**Authors:** Maria Anurova, Aleksey Kuzmin, Aleksey Vorobev, Nataliya Feldman, Elena Zinurova, Andrey Aleshkin

**Affiliations:** 1 Sechenov First Moscow State Medical University, 8-2 Trubetskaya Street, Moscow 119048, Russia; anurova_m_n@staff.sechenov.ru (M.A.); n_feldman@mail.ru (N.F.); lenazinurva@yandex.ru (E.Z.); 2 Federal Budgetary Institution of Science «Moscow Research Institute of Epidemiology and Microbiology named after G. N. Gabrichevsky», Federal Service for Surveillance on Consumer Protection and Human Health (Rospotrebnadzor), 10 Admirala Makarova Street, Moscow 125212, Russia; vorobjew.alex2010@yandex.ru; 3 Orphan-Bio LLC, Triumphalny Proezd, 1, Sirius 354340, Russia; andreialeshkin@googlemail.com

**Keywords:** bacteriophages, pharmacokinetics, oral route of administration, rectal route of administration, Gratia method, PCR

## Abstract

The effect of administration routes (oral and rectal) on the pharmacokinetics of Salmonella phage SE40 and Escherichia phage V18 was studied. To detect phages in biomaterial (blood, urine, and feces), the Spot-test, Gratia, and double-nested polymerase chain reaction methods were used. Systemic action of the studied phages with both routes of administration, beginning 15–30 min after administration, was demonstrated. The phages persisted for up to 24 h after both oral and rectal administration. However, the concentration in the blood was higher after oral administration, while concentrations in urine and feces were higher after rectal administration. The need to protect phages from the acidic contents of the stomach was confirmed.

## 1. Introduction

Numerous studies conducted by Russian and foreign scientists demonstrate the possibility of using bacteriophages as natural antimicrobial agents to fight bacterial infections in humans and animals, including those caused by antibiotic-resistant strains [1,2,3,4,5,6,7,8,9,10,11,12,13,14]. Bacteriophages as drugs are historically used in the form of solutions for oral administration [12]. On the Russian pharmaceutical market, 16 bacteriophage drugs manufactured by the Scientific and Production Association for Immunological Preparations “Microgen” are registered in the form of solutions for oral administration, external or rectal use, tablets, and coated tablets, both based on certain types of bacteriophages and their combinations for the treatment and prevention of acute intestinal infections and decompensated forms of dysbiosis, as well as pyoinflammatory diseases of bacterial genesis.

Over the past decade, significant efforts have been made in the development of new enteral formulations of bacteriophages. Studies on the oral administration of phages have been conducted. Bacteriophages against *E. coli* for the treatment of gastrointestinal diseases were assessed in phase I placebo-controlled trials among healthy adults in Switzerland (2005 [15]) and Bangladesh (2012 [16]), and among healthy and diseased children in Bangladesh (2017 [17]). During 2017–2018, a randomized, placebo-controlled, double-blind clinical trial was conducted to evaluate the polyvalent phage preparation “Pyophage” for the treatment of urinary tract infections in patients who had undergone transurethral resection of the prostate (clinical trial No. NCT03140085) [18]. In 2020, a study at Westmead Hospital, Australia, investigated the intravenous administration of the phage cocktail AB-SA01, which includes three different bacteriophages produced by AmpliPhi Biosciences, USA, for the treatment of infective endocarditis and sepsis caused by Staphylococcus [19]. AmpliPhi Biosciences received FDA approval to conduct trials of an intravenous phage preparation for the treatment of purulent wounds and skin infections caused by methicillin-resistant *S. aureus* strains.

Although phages have been used in medicine for quite some time, there are insufficient data on their pharmacokinetic profiles. Lytic phages are biological organisms that replicate in the presence of susceptible bacteria and have a complex pharmacokinetic profile, which is primarily influenced by the route of administration, the size of the phages, and the location of the infection [20].

Bacteriophages are capable of circulating and accumulating in various organs and tissues. After introduction into the body, phages were detected in the blood, bronchoalveolar lavage fluid, and feces, as well as in various organs, including the lungs, heart, liver, kidneys, and spleen. They have also been detected in brain tissue, indicating their ability to cross the blood–brain barrier [21].

The oral route is the most convenient and popular method for delivering drugs to the human body; however, the bioavailability of phages following oral administration is often low. Several studies have shown that intraperitoneal administration allows phages to enter the systemic circulation 3 h earlier than oral administration. Some experimental data indicate that approximately 50% of oral preparations were ineffective when taken orally [1]. It is known that significant loss of phage titer occurs with oral administration due to their low stability in the acidic environment of the stomach [22,23]. For this reason, antacids are often used or recommended to help phages pass through the stomach [1].

However, there is also evidence of successful oral administration of bacteriophages in humans and animals. For example, after oral administration of the Klebsiella phage Kp_Pokalde_002 to mice, the blood titer peaked after 8 h and then declined over the course of 48 h [24]. When a cocktail of 9 T4-like coliphages was administered orally to 15 healthy adults, they were detected in the blood and feces [16], and similar results have been obtained when using phages to treat *Escherichia coli* infection in children [17].

Phages can accumulate in the liver and spleen; it is believed that Kupffer cells in the liver and macrophages in the spleen phagocytose the phages. It is hypothesized that non-specific clearance by the mononuclear phagocyte system is the most likely mechanism by which phages are removed from the bloodstream [25]. There is considerable individual variation in the renal excretion of phages; these differences may be related to the host’s age, health status, type of infection, renal function, and variability in phage transcytosis, among other factors, and have not yet been fully studied [26].

There are no data on the pharmacokinetics and pharmacodynamics of phages following rectal administration. Given the data on the efficacy of phage therapy when applied to mucous membranes, rectal administration of these drugs is relevant. This route of administration could achieve systemic effects, high speed of drug delivery into the systemic circulation, and be convenient for patients with conditions like gastrointestinal infections accompanied by vomiting (which make oral administration difficult), as well as for children and elderly patients [22].

For a justified choice of the dosage form, reliable data on the impact of the administration route on pharmacokinetics is essential. This study is the first to compare the pharmacokinetic parameters of bacteriophages administered orally and rectally.

Thus, the aim of this work was a comparative study of the pharmacokinetics of the Salmonella phage SE40 and Escherichia phage V18 after oral and rectal administration.

## 2. Materials and Methods

Bacteriophages that lyse *Salmonella enteritidis* and *E. coli* were used (Table 1). Bacteriophage strains SE40 and V18 were isolated from fecal samples collected from patients at the Clinical Center of the Moscow Research Institute of Epidemiology and Microbiology named after G. N. Gabrichevsky. The strains were characterized by a number of physicochemical and molecular biological characteristics; the results are presented in Table 1. Highly virulent phages, stable for a long time (more than 1 year), were selected and used for the treatment of intestinal infections and are therefore suitable for various enteral routes of administration. The lytic spectrum of the strains was determined using 60 corresponding bacterial strains isolated from patients. The selected bacteriophages exhibited activity exceeding 80%.

To study the pharmacokinetics, phages were administered as a solution for oral administration and rectal suppositories. By solution we mean a dosage form, which is a suspension of bacteriophages in an isotonic solution of sodium chloride. The composition of the administered dosage forms is presented in Table 2.

The experiment included 26 chinchilla rabbits (males) weighing from 3.5 to 4 kg. Before the experiment, the rabbits were quarantined for two weeks. Clinical and laboratory screening was performed for the presence of enterotoxemia, salmonellosis, colibacillosis, pasteurellosis, ringworm, coccidiosis, and ear mites. The animals were cared for and kept under the recommendations and requirements of Directive 2010/63/EU of the European Parliament and of the EU Council of 22 September 2010 on the protection of animals used for scientific purposes. All animal experiments were approved by the ethics committee of G.N. Gabrichevsky Moscow Research Institute for Epidemiology and Microbiology.

Before the administration of a cocktail of bacteriophages in the form of a solution, the pH of the gastric juice of 12 rabbits was neutralized by oral administration of 3 mL of the commercial drug Almagel^®^ (Teva, Moscow, Russia) 30 min before the start of the experiment. 30 min after the administration of Almagel^®^, each animal was orally administered 3 mL of a cocktail of bacteriophages. The pH of the stomach was not neutralized in three rabbits. To demonstrate the need for pre-administration of an antacid, two rabbits were administered a solution containing a bacteriophage cocktail without prior neutralization of gastric pH.

Another 12 rabbits were administered a suppository into the rectum to a depth of about 5 cm; the rectum was clamped for 15–20 min, after which samples were taken.

Samples of blood, feces, and urine were collected from animals over 24 h. Twenty hours before the start of the experiment, the feeding of animals was stopped; 1 h before the procedure, the water bottles were removed. Blood was taken from the ear vein after 15, 30, 45, and 75 min, 3 h, and 24 h. Feces and urine were collected at 1, 3, 4.5, 6, and 24 h. All urine and feces were collected during the 24 h of observation.

Blood was taken from the ear veins of rabbits into tubes with ethylenediaminetetraacetic acid (EDTA), after which the blood was used without pretreatment. Urine was shaken with chloroform at a ratio of 1:10 for 15 min, then centrifuged for 30 min at 5000 rpm (Eppendorf MiniSpin centrifuge, Eppendorf AG, Hamburg, Germany), and the upper liquid layer was collected for analysis. Feces were triturated with 10–15 mL of saline, chloroform was added in a ratio of 1:10 and shaken for 15 min, after which the mixture was centrifuged for 30 min at 5000 rpm, and the upper liquid layer was collected for analysis.

In the course of pharmacokinetic studies, to confirm the presence of phage particles in clinical samples, the blood, urine, and feces of rabbits before and after a single administration of bacteriophages were sequentially investigated by microbiological spot-test, Gratia, and molecular genetic double-nested PCR methods [10].

Spot-test is a method of applying phage to the lawn of a bacterial culture [10]. 1.5% nutrient agar was poured into Petri dishes. After the agar had solidified and dried, 0.1 mL of a 16–18 h broth culture of microorganisms sensitive to bacteriophages in the test filtrate was applied to Petri dishes and spread with a spatula over the entire surface to obtain uniform continuous growth of the culture. After the culture had been absorbed and the plates were dry, the test filtrate was applied dropwise (spot-test). After drying, the plates were turned upside down and placed in a thermostat at 37 °C for 18–24 h. On the next day, the results were recorded as the presence or absence of a “lysis spot”.

The Gratia method (the method of agar layers) is based on the introduction of various dilutions of the titrated bacteriophage into the corresponding culture of bacteria and inoculation onto a solid nutrient medium to obtain negative colonies (lysis zones or plaques) of the bacteriophage [9]. On the eve of the experiment, culture media were prepared: 1.5% nutrient agar was poured into sterile Petri dishes (25 mL each); sterile nutrient broth (4.5 mL each) and 0.7% semi-solid agar (2.5 mL each) were prepared in tubes. On the day of the experiment, serial dilutions were prepared in test tubes from a liquid containing a titratable bacteriophage. Then, 1 mL of the corresponding dilution of the studied bacteriophage was added to a test tube with 0.7% semi-solid agar melted and cooled to 50–52 °C, mixed slightly, then 0.1–0.2 mL of a 10^9^ CFU/mL suspension of a culture sensitive to the bacteriophage was added, mixed slightly again, and the contents of the tube were poured into a dish with nutrient agar (second layer). After the medium cooled down, the dishes were incubated in a thermostat at 37 °C for 18–20 h. Phage titer was determined by counting the number of negative colonies on parallel plates and multiplying the arithmetic mean by the dilution index.

Double-nested PCR: isolation of bacteriophage DNA from blood was performed using a K-sorb isolation kit (LLC “NPF Syntol”, Moscow, Russia). Each reaction used 1 μL of isolated DNA. Highly sensitive detection of phage DNA fragments was carried out using double-nested PCR. Reaction mixtures were prepared using the reagents included in the recombinant Taq DNA polymerase kit (Fermentas, Vilnius, Lithuania). For the reactions, the oligonucleotide sequences shown in Table 3 were used. The amplification result was detected by performing horizontal electrophoresis in 1% agarose gel.

Descriptive statistics on the lg titers of the PFU of bacteriophages are presented as the average (median) value and interquartile range of values characterizing the error of the mean. The normality of the distribution of values at each point was checked using the Shapiro–Wilk test, the *p*-value of which for all points was below 0.05, which indicates the absence of a normal distribution (statistical analysis package for Microsoft Excel 2007 (Microsoft Corporation, Redmond, WA, USA)).

## 3. Results

8 rabbits were orally administered a bacteriophage cocktail in the form of a solution. Six rabbits were given pre-neutralization of gastric pH with an antacid, while two were not. It was decided to preliminarily introduce an antacid drug since it is known that many bacteriophages, when taken orally, can lose activity under the action of gastric juice and in the acidic environment of the stomach. Both SE40 and V18 phages under study are unstable at pH less than 5 [9]. 6 rabbits received placebo. The selected samples of blood, urine, and feces were first studied by spot-test and, after obtaining positive results, analyzed by the Gratia method. This technique was applied to all samples.

Another 12 rabbits were administered suppositories with the same phage cocktail. 6 rabbits were administered suppositories with bacteriophages, and 6 were given placebo. The results of determining the concentration of bacteriophages in biomaterials are presented in Table 4, Table 5, Table 6 and Table 7. No bacteriophages were detected in the urine or blood of the placebo group following oral administration.

The next stage of pharmacokinetic studies was the identification of phage DNA fragments in the blood of rabbits after administration of the solution and suppositories. To identify fragments of phage DNA in clinical material, the nested PCR method with two pairs of specific primers was used. The resolution of the method is 1–10 copies/μL. In the presence of a positive signal in the first round, the signal intensity increases in the next round, which indicates the specificity of the reaction. In the absence of a signal in the first round, the use of nested PCR allows, in the presence of DNA copies in the template sample, to obtain a signal in the second round.

**Figure 1:**
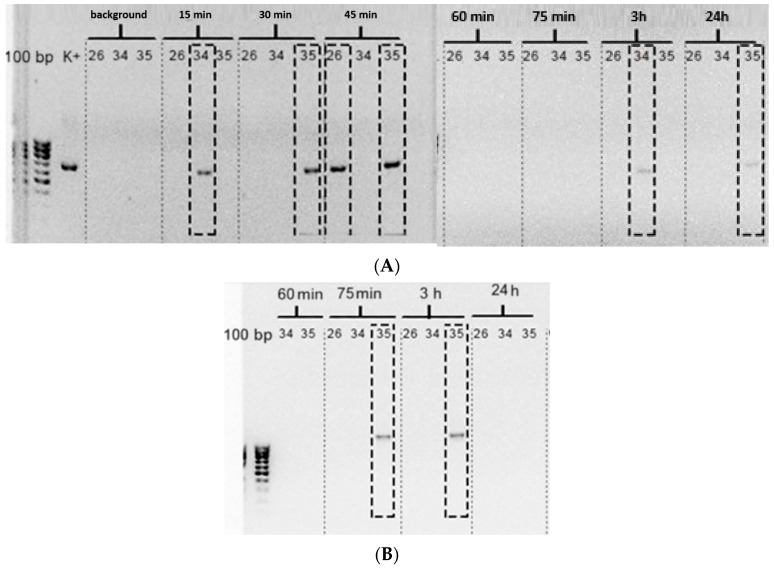
Dynamics of the presence of SE40 (**A**) and V18 (**B**) bacteriophages DNA in the blood of rabbits after administration of the solution.

**Figure 2:**
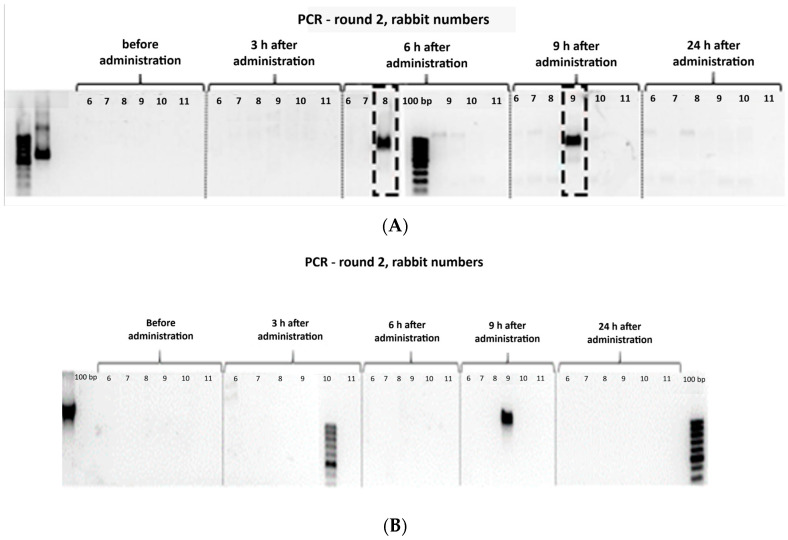
Dynamics of the presence of SE40 (**A**) and V18 (**B**) bacteriophages DNA in the blood of rabbits after administration of suppositories.

In the first round, template DNA is used, then the second round is carried out with the resulting amplicon, and with the next round, the specificity of the reaction increases. The data obtained are presented in Figure 1 and Figure 2, positive results are highlighted with a dotted line.

## 4. Discussion

For the treatment of acute intestinal infections caused by *S. enteritidis* and *E. coli*, it is important to introduce antibacterial agents into the intestines. In pathological conditions, these bacteria inhabit the small intestine and, in severe cases, the large intestine. To provide therapeutic effects, bacteriophages must reach the site of inflammation, where they will actively multiply and suppress the pathogenic flora. When drugs are taken orally, they are exposed to gastric juices and undergo presystemic elimination, which can significantly reduce the effectiveness of therapy. When administered rectally, drugs bypass the liver to a greater extent, and presystemic metabolism is significantly lower than when administered orally. In addition, *E. coli* and *Salmonella* infections are often accompanied by vomiting and nausea, which makes oral administration difficult, as well as diarrhea, which can pose challenges for rectal administration. As with most drugs, administration route and dosage are key factors that affect phage circulation time in vivo.

**Figure 3:**
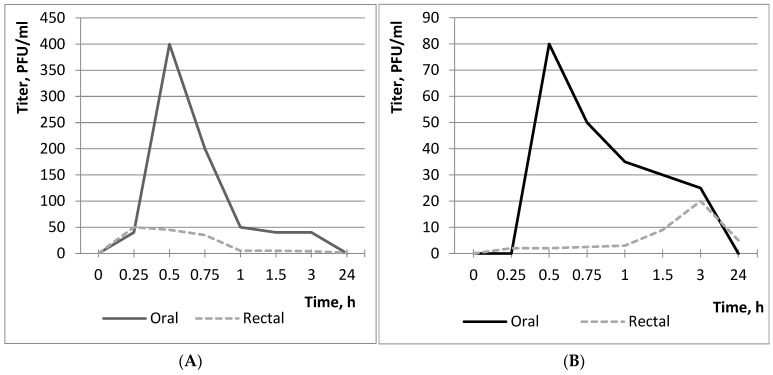
Dynamics of the concentration of SE40 (**A**) and V18 (**B**) bacteriophages in the blood of rabbits after oral and rectal administration of dosage forms.

After the introduction of the bacteriophage cocktail, the drug substances reach the systemic circulation through both oral and rectal routes of administration (Figure 3). The maximum concentration of bacteriophages SE40 and V18 after oral administration is observed after 30 min; the phages persist in the blood for more than 3 h, but after 24 h they are not detected in the blood. The maximum concentration of bacteriophages with rectal administration is also reached after 30–45 min; however, phages are found in the systemic circulation one day after administration. The concentration of bacteriophage SE40 30 min after administration is more than 8 times higher with oral administration than with rectal administration (4.0 × 10^2^ PFU/mL when administered orally vs. 4.5 × 10^1^ PFU/mL when administered rectally), and the concentration of phage V18 is forty times higher (1.6 × 10^3^ PFU/mL when administered orally vs. 2.0 × 10^3^ PFU/mL when administered rectally). When bacteriophages are administered orally without first neutralizing the stomach pH, phages are detected in the blood only at the first two time points and in smaller quantities than when an antacid is used, confirming the instability of phage particles in the acidic environment of the stomach. Data on instability in the stomach and upper parts of the small intestine, associated with low pH levels, are also confirmed by other researchers [1,27]. In the work of Arne Echterhof and co-authors, it was demonstrated that when radiolabeled phage strains PAML-31-1, OMKO1, and Luz24, lytic to drug-resistant *Pseudomonas aeruginosa*, were administered orally to normal young adult CD-1 mice, their concentration in the blood in vivo became detectable [28]. According to several studies, the maximum number of phages in the blood is reached in the first hours after administration, and then their number decreases after 12–24 h. The time to reach the peak concentration of phages in the blood may be influenced by their size and the presence of host bacteria [24,29].

**Figure 4:**
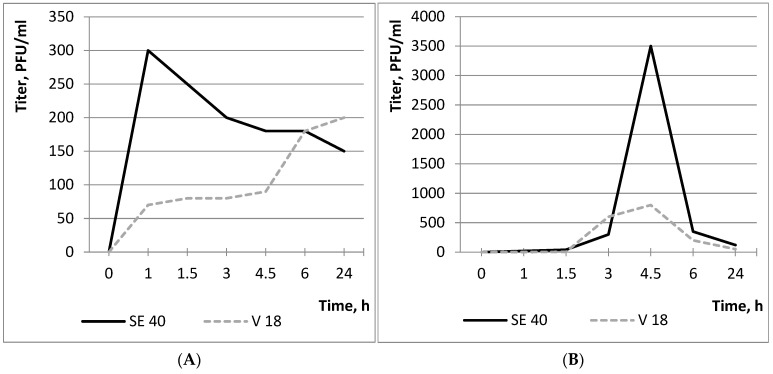
Dynamics of the concentration of SE40 and V18 bacteriophages in the urine of rabbits after oral (**A**) and rectal (**B**) administration of dosage forms.

The dynamics of the presence of bacteriophages in urine are as follows: when taken orally, phages in urine begin to be detected one hour after administration (first observation point) and reach the first peak of concentration (3.0 × 10^2^ PFU/mL for SE40, 7.0 × 10^1^ PFU/mL for V18); then the concentration of phages decreases slightly and increases (only for phage V18) after 24 h (1.5 × 10^2^ PFU/mL for SE40, 2.0 × 10^2^ PFU/mL for V18). With rectal administration of phages, they also begin to be detected one hour after administration; their concentration in urine reaches a maximum 4.5 h after administration (3.5 × 10^3^ PFU/mL for SE40, 8.0 × 10^2^ PFU/mL for V18), then the concentration decreases and after 24 h is 1.2 × 10^2^ PFU/mL for SE40 and 5.0 × 10^1^ PFU/mL for V18. The maximum concentration of SE40 in urine with rectal administration is more than 10 times higher than with oral administration, and for phage V18 it is more than 4 times higher. The dynamics of phage excretion in urine for both administration routes are illustrated in Figure 4.

Renal clearance is the general mechanism of elimination for most common small molecule drugs, but data on renal clearance of phages and their detection in urine vary [1,30]. In our studies, we showed that phages are detected in urine after both oral and rectal administration.

**Figure 5:**
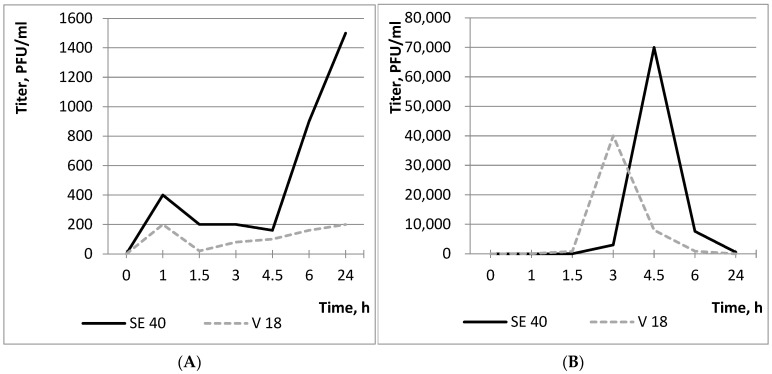
Dynamics of the concentration of SE 40 and V 18 bacteriophages in the feces of rabbits after oral (**A**) and rectal (**B**) administration of dosage forms.

Bacteriophages begin to be detected in feces one hour after administration of the dosage forms, except for SE40, which, when administered rectally, is detected only after 1.5 h. The maximum concentration of bacteriophages in feces after oral administration is reached after 24 h: SE40—1.5 × 10^3^ PFU/mL, and V18—2.0 × 10^2^ PFU/mL. With rectal administration, a different dynamic is observed: the maximum is reached at 4.5 h and is for SE40—7.5 × 10^4^ PFU/mL, and V18—8.0 × 10^3^ PFU/mL, which is 30 and 40 times higher than with oral administration for SE40 and V18, respectively; then the concentration of phages decreases, and SE40 is not detected in feces after 24 h. The comparative dynamics of phage excretion in feces for both administration routes are presented in Figure 5.

The work carried out at the Nestlé Research Center demonstrates the possibility of treating diarrhea caused by *E. coli* using T4-like phages. The phages selected by the researchers survived passage through the gastrointestinal tract of adult mice. The number of phages in the feces was quite high and approximately corresponded to the titer of phages administered orally. However, the authors believe that such a high concentration may be associated with the proliferation of the phages in the endogenous intestinal population of *E. coli* [31]. This correlates with the data we obtained, except that there were no host bacteria in the intestines of the rabbits used, so the phage titers were lower. Confirmation that phages successfully pass through the gastrointestinal tract comes from the widely available data on the treatment of acute intestinal infections [32,33,34,35].

Highly sensitive detection of phage DNA fragments in the blood of animals is shown in Figure 1 and Figure 2 in the form of horizontal electrophoresis of products (amplicons) of the 2nd round of PCR, confirming the presence of bacteriophage DNA in the blood of rabbits from 1000 to 10,000 copies per ml. The probability of detecting phage DNA (the maximum number of copies of various phage strains) increases within 1.5–3 h after administration of a solution with a phage cocktail and within 6–9 h after administration of suppositories with phages.

The obtained data on the pharmacokinetics of phages after oral administration are consistent with a number of publications. Gut transit of active bacteriophages is typically assessed by the detection of active phages in feces after oral application. Bacteriophages can commonly travel through the gastrointestinal tract. Specifically, this was demonstrated in human safety studies and in animal models, including mice, rats, chickens, quails, calves, pigs, and sheep. Although bacteriophages pass through the alimentary tract, their recovery from feces can be different [30]. Low phage penetration and dilution in the blood volume may result in undetectable phage titers, even if rare phage particles are able to penetrate organs. These phages, however, if they reach a site of bacterial infection, can propagate on susceptible bacteria, increasing the phage amount in situ. Orally delivered phages can be effective in phage therapy in many body sites distant from the alimentary tract [36,37]. There are very little data on the pharmacokinetics of phages after rectal administration, and they are presented in only a few publications, so it is not possible to compare them [20]. However, there is clinical evidence of successful rectal use of phages. The publication describes the successful treatment of patients with chronic prostatitis caused by *E. coli* with rectal administration of phages twice a day (at a dose of 10^8 to 10^9 PFU per dose for approximately 1 month). Similarly, a 60-year-old kidney transplant patient was successfully treated for a chronic urinary tract infection caused by *K. pneumoniae* [38].

Furthermore, the reliability of data on the quantitative content of phages in a given locus is affected by the detection method. The most commonly used methods, agar layers and PCR, have limitations in sensitivity and measurement error. In complex samples, specific pre-treatment steps are needed to enrich viral particles for accurate results [39].

## 5. Conclusions

Against the backdrop of the rapid spread of multidrug-resistant bacteria that cause infections, there is an urgent need to develop alternative therapeutic approaches, among which phage therapy stands out. Unfortunately, progress in studying the pharmacokinetics of phages has lagged behind the growing need for their use. When developing bacteriophage-based therapeutics, the choice of route of administration is critical, and this study clearly demonstrated its impact on pharmacokinetic characteristics. When administered rectally and orally, phages have a potential systemic effect. They are found in the blood 15–30 min after the administration of dosage forms; the concentration of phages in the blood when taken orally is higher than when taken rectally, but when used rectally, phages are found in the blood for up to 24 h, which is confirmed by the double-nested PCR method. Phage concentrations in urine and feces are higher with rectal administration. Although the oral route is the most convenient method of administering drugs to humans, the bioavailability of phages administered this way is typically low. Nevertheless, oral administration remains a promising strategy for treating bacterial infections of the gastrointestinal tract, but requires the use of technological strategies to prevent phage inactivation in the stomach. Meanwhile, rectal administration may be promising for the systemic delivery of phages. Studying the pharmacokinetic properties of lytic phages is a fundamental step toward their successful implementation in clinical practice.

## Figures and Tables

**Table 1:** Characteristics of bacteriophages used.

	Bacteriophage Name	*Salmonella phage* SE40	*Escherichia phage* V18
Characteristic			
Taxonomic affiliation		Order *Caudovirales*; Family *Siphoviridae*; Subfamily *Guernseyvirinae*; Genus *Jerseyvirus*	Order *Caudovirales*; Family *Myoviridae*; Subfamily *Vequintavirinae*; Genus *V5virus*
GenBank registration number		KY626163.1	KY683736.1
Electron microscope image with superimposed scale 100 nm		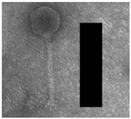	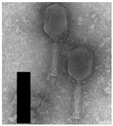
Temperature resistance		no more than 80 °C	no more than 60 °C
pH resistance		5.2–9.6	7.0–9.6
DNA size, kbp		29	129
Phage length (head, tail, and baseplate), nm		122	146
The diameter (largest width) of the head, nm		35	46

**Table 2:** Composition of samples for oral (solution) and rectal administration (suppositories).

	Composition	SE40 Bacteriophage Titer	V18 Bacteriophage Titer	Excipients
Type of Dosage Form				
Placebo for oral administration		-	-	Water for injection
Solution		3.0 × 10^7^ PFU/mL	7.0 × 10^7^ PFU/mL	Water for injection
Placebo for rectal administration (phage-free suppositories)		-	-	Polysorbate 80 0.037 g; Witepsol^®^ W 35 (IOI Oleo GmbH, Hamburg, Germany): Witepsol^®^ H15 (1:1) (IOI Oleo GmbH, Hamburg, Germany) up to 1.22 g
Suppositories		7.0 × 10^7^ PFU/supp.	6 × 10^7^ PFU/supp.	Polysorbate 80 0.037 g; Witepsol^®^ W 35 (IOI Oleo GmbH, Hamburg, Germany): Witepsol^®^ H15 (1:1) (IOI Oleo GmbH, Hamburg, Germany) up to 1.22 g

PFU—plaque-forming unit.

**Table 3:** Oligonucleotide sequences for detecting DNA fragments of phages.

Oligonucleotide Name	Oligonucleotide Purpose	Nucleotide Sequence, 5′-3′	Description
Target—SE40 bacteriophage DNA			
BPhSE1_fw1	Direct primer	tgcagatatcgactacgcc	1st PCR round
BPhSE1_rv1	Reverse primer	tcaacgcctgtacatccc	
BPhSE1_fw2	Direct primer	gtgatgctggtttctccc	2nd PCR round
BPhSE1_rv2	Reverse primer	ctttgccctttagcttccg	
Target–V18bacteriophage DNA			
BPhEc2_fw1	Direct primer	cagaaggacgttgatccgg	1st PCR round
BPhEc2_rv1	Reverse primer	ttaacctcggaacccagcc	
BPhEc2_fw2	Direct primer	atacgaacgaagtgcaggg	2nd PCR round
BPhEc2_rv2	Reverse primer	tggtggaagatggtttcgc	

**Table 4:** The presence of the studied bacteriophages in the blood of rabbits after oral administration of dosage forms (Gratia method).

	Bio- Material Type	Bacteriophage Titer in Blood, PFU/mL					
Time, h		Placebo		Solution Without Prior Neutralization of Gastric pH		Solution	
Background		-	-	-	-	-	-
0.25		-	-	1.0 ± 0.02 × 10^1^	1.0 ± 0.03 × 10^1^	4.0 ± 0.5 × 10^1^	-
0.5		-	-	2.0 ± 0.08 × 10^2^	1.0 ± 0.02 × 10^1^	4.0 ± 0.7 × 10^2^	8.0 ± 0.7 × 10^1^
0.75		-	-	-	-	2.0 ± 0.2 × 10^2^	5.0 ± 0.5 × 10^1^
1.0		-	-	-	-	5.0 ± 0.6 × 10^1^	3.5 ± 0.4 × 10^1^
1.5		-	-	-	-	4.0 ± 1.0 × 10^1^	3.0 ± 0.5 × 10^1^
3.0		-	-	-	-	4.0 ± 1.5 × 10^1^	2.5 ± 0.7 × 10^1^
24.0		-	-	-	-	-	-
Bacteriophage strains		SE40	V18	SE40	V18	SE40	V18

“-”—no phage particles.

**Table 5:** The presence of the studied bacteriophages in the blood of rabbits after rectal administration of dosage forms (Gratia method).

	Bio- Material Type	Bacteriophage Titer in Blood, PFU/mL			
Time, h		Placebo		Suppositories	
Background		-	-	-	-
0.25		-	-	5.0 ± 1.5 × 10^1^	2.0 ± 0.5 × 10^0^
0.5		-	-	4.5 ± 1.2 × 10^1^	2.0 ± 0.9 × 10^0^
0.75		-	-	3.5 ± 0.8 × 10^1^	2.5 ± 0.6 × 10^0^
1.0		-	-	5.0 ± 1.0 × 10^0^	3.0 ± 0.9 × 10^0^
1.5		-	-	5.0 ± 0.5 × 10^0^	9.0 ± 1.6 × 10^0^
3.0		-	-	4.0 ± 0.7 × 10^0^	2.0 ± 0.9 × 10^1^
24.0		-	-	1.0 ± 0.4 × 10^0^	5.0 ± 1.1 × 10^0^
Bacteriophage strains		SE40	V18	SE40	V18

“-”—no phage particles.

**Table 6:** The presence of the studied bacteriophages in the urine of rabbits after oral and rectal administration of dosage forms (Gratia method), PFU/mL.

	Bio- Material Type	Bacteriophage Titer (PFU/mL) in							
		Oral				Rectal			
Time, h		Solution Without Prior Neutralization of Gastric pH		Solution		Placebo		Suppositories	
Background		-	-	-	-	-	-	-	-
1.0		-	-	3.0 ± 1.6 × 10^2^	7.0 ± 0.6 × 10^1^	-	-	2.0 ± 0.5 × 10^1^	2.0 ± 0.4 × 10^0^
1.5		-	-	2.5 ± 0.9 × 10^2^	8.0 ± 0.9 × 10^1^	-	-	4.0 ± 1.6 × 10^1^	5.0 ± 1.2 × 10^0^
3.0		-	5.0 ± 0.2 × 10^1^	2.0 ± 1.0 × 10^2^	8.0 ± 0.8 × 10^1^	-	-	3.0 ± 2.0 × 10^2^	6.0 ± 0.8 × 10^2^
4.5		1.0 ± 0.4 × 10^1^	-	1.8 ± 0.5 × 10^2^	9.0 ± 1.7 × 10^1^	-	-	3.5 ± 1.5 × 10^3^	8.0 ± 1.3 × 10^2^
6.0		-	-	1.8 ± 1.4 × 10^2^	1.8 ± 1.1 × 10^2^	-	-	3.5 ± 1.5 × 10^2^	2.0 ± 1.2 × 10^2^
24.0		-	-	1.5 ± 0.5 × 10^2^	2.0 ± 0.7 × 10^2^	-	-	1.2 ± 0.6 × 10^2^	5.0 ± 0.5 × 10^1^
Bacteriophage strains		SE40	V18	SE40	V18	SE40	V18	SE40	V18

“-”—no phage particles.

**Table 7:** The presence of the studied bacteriophages in the feces of rabbits after oral and rectal administration of dosage forms (Gratia method), PFU/mL.

	Bio- Material Type	Bacteriophage Titer (PFU/mL) in							
		Oral				Rectal			
Time, h		Solution Without Prior Neutralization of Gastric pH		Solution		Placebo		Suppositories	
Background		-	-	-	-	-	-	-	-
1.0		-	-	4.0 ± 1.1 × 10^2^	2.0 ± 0.5 × 10^2^	-	-	-	5.0 ± 1.1 × 10^1^
1.5		-	-	2.0 ± 2.2 × 10^2^	2.0 ± 1.4 × 10^1^	-	-	5.0 ± 1.6 × 10^0^	8.0 ± 1.9 × 10^2^
3.0		-	-	2.0 ± 2.0 × 10^2^	8.0 ± 2.5 × 10^1^	-	-	3.0 ± 1.6 × 10^3^	4.0 ± 2.5 × 10^4^
4.5		-	-	1.6 ± 1.8 × 10^2^	1.0 ± 1.9 × 10^2^	-	-	7.0 ± 3.8 × 10^4^	8.0 ± 3.5 × 10^3^
6.0		-	2.0 ± 1.0 × 10^1^	9.0 ± 2.9 × 10^2^	1.6 ± 1.8 × 10^2^	-	-	7.6 ± 4.2 × 10^3^	9.0 ± 2.2 × 10^2^
24.0		4.0 ± 1.4 × 10^1^	-	1.5 ± 2.9 × 10^3^	2.0 ± 2.3 × 10^2^	-	-	5.0 ± 2.7 × 10^2^	-
Bacteriophage strains		SE40	V18	SE40	V18	SE40	V18	SE40	V18

“-”—no phage particles.

## Data Availability

This article contains the original contributions presented in the study. For any further inquiries, please contact the corresponding author.

## References

[1] Dąbrowska K., Abedon S.T. (2019). Pharmacologically Aware Phage Therapy: Pharmacodynamic and Pharmacokinetic Obstacles to Phage Antibacterial Action in Animal and Human Bodies. Microbiol. Mol. Biol. Rev..

[2] Malik D.J., Sokolov I.J., Vinner G.K., Mancuso F., Cinquerrui S., Vladisavljevic G.T., Clokie M.R.J., Garton N.J., Stapley A.G.F., Kirpichnikova A. (2017). Formulation, stabilization and encapsulation of bacteriophage for phage therapy. Adv. Colloid Interface Sci..

[3] Maciejewska B., Olszak T., Drulis-Kawa Z. (2018). Applications of bacteriophages versus phage enzymes to combat and cure bacterial infections: An ambitious and also a realistic application?. Appl. Microbiol. Biotechnol..

[4] Payne R.J., Phil D., Jansen V.A. (2000). Phage therapy: The peculiar kinetics of self-replicating pharmaceuticals. Clin. Pharmacol. Ther..

[5] Brüssow H. (2005). Phage therapy: The *Escherichia coli* experience. Microbiology.

[6] Abedon S.T., Kuhl S.J., Blasdel B.G., Kutter E.M. (2011). Phage treatment of human infections. Bacteriophage.

[7] Bardina C., Spricigo D.A., Cortés P., Llagostera M. (2012). Significance of the bacteriophage treatment schedule in reducing *Salmonella* colonization of poultry. Appl. Environ. Microbiol..

[8] Colom J., Cano-Sarabia M., Otero J., Aríñez-Soriano J., Cortés P., Maspoch D., Llagostera M. (2017). Microencapsulation with alginate/CaCO_3_: A strategy for improved phage therapy. Sci. Rep..

[9] Albac S., Medina M., Labrousse D., Hayez D., Bonnot D., Anzala N., Laurent F., Ferry T., Dublanchet A., Chavanet P. (2020). Efficacy of Bacteriophages in a *Staphylococcus aureus* Nondiabetic or Diabetic Foot Infection Murine Model. Antimicrob. Agents Chemother..

[10] Tan C.S., Aqiludeen N.A., Tan R., Gowbei A., Mijen A.B., Santhana Raj L., Ibrahim S.F. (2020). Could bacteriophages isolated from the sewage be the solution to methicillin-resistant *Staphylococcus aureus*?. Med. J. Malays..

[11] Bleibtreu A., Fevre C., Robert J., Haddad E., Caumes E., Lantieri L., Peyre M. (2020). Combining bacteriophages and dalbavancin for salvage therapy of complex *Staphylococcus aureus* extradural empyema. Med. Mal. Infect..

[12] Pyzik E., Radzki R.P., Urban-Chmiel R. (2021). Experimental phage therapies in companion animals with historical review. Curr. Rev. Clin. Exp. Pharmacol..

[13] Gupta T., Kanchan, Bhagyawant S.S. (2023). ‘Bacteriophage therapy’ an emerging cure for bacterial disease. Int. J. Curr. Pharm. Res..

[14] Duraisamy N., Nachimuthu R., Vaithilingam K., Pandiyan R., Ebenezer King S., Velu Rajesh K. (2015). Distribution, isolation and characterization of lytic bacteriophages against multi-drug resistant and extended-spectrum of î^2^-lactamase producing pathogens from hospital effluents. Asian J. Pharm. Clin. Res..

[15] Bruttin A., Brüssow H. (2005). Human volunteers receiving *Escherichia coli* phage T4 orally: A safety test of phage therapy. Antimicrob. Agents Chemother..

[16] Sarker S.A., McCallin S., Barretto C., Berger B., Pittet A.C., Sultana S., Krause L., Huq S., Bibiloni R., Bruttin A. (2012). Oral T4-like phage cocktail application to healthy adult volunteers from Bangladesh. Virology.

[17] Sarker S.A., Berger B., Deng Y., Kieser S., Foata F., Moine D., Descombes P., Sultana S., Huq S., Bardhan P.K. (2017). Oral application of *Escherichia coli* bacteriophage: Safety tests in healthy and diarrheal children from Bangladesh. Environ. Microbiol..

[18] Leitner L., Sybesma W., Chanishvili N., Goderdzishvili M., Chkhotua A., Ujmajuridze A., Schneider M.P., Sartori A., Mehnert U., Bachmann L.M. (2017). Bacteriophages for treating urinary tract infections in patients undergoing transurethral resection of the prostate: A randomized, placebo-controlled, double-blind clinical trial. BMC Urol..

[19] Petrovic Fabijan A., Lin R.C.Y., Ho J., Maddocks S., Ben Zakour N.L., Iredell J.R., Westmead Bacteriophage Therapy Team (2020). Safety of bacteriophage therapy in severe *Staphylococcus aureus* infection. Nat. Microbiol..

[20] Siopi M., Skliros D., Paranos P., Koumasi N., Flemetakis E., Pournaras S., Meletiadis J. (2024). Pharmacokinetics and pharmacodynamics of bacteriophage therapy: A review with a focus on multidrug-resistant Gram-negative bacterial infections. Clin. Microbiol. Rev..

[21] Nang S.C., Lin Y.W., Petrovic Fabijan A., Chang R.Y.K., Rao G.G., Iredell J., Chan H.K., Li J. (2023). Pharmacokinetics/pharmacodynamics of phage therapy: A major hurdle to clinical translation. Clin. Microbiol. Infect..

[22] Khanal D., Chang R.Y.K., Hick C., Chan H.K. (2021). Enteric-coated bacteriophage tablets for oral administration against gastrointestinal infections. Int. J. Pharm..

[23] Vinner G.K., Malik D.J. (2018). High precision microfluidic microencapsulation of bacteriophages for enteric delivery. Res. Microbiol..

[24] Dhungana G., Nepal R., Regmi M., Malla R. (2021). Pharmacokinetics and Pharmacodynamics of a Novel Virulent Klebsiella Phage Kp_Pokalde_002 in a Mouse Model. Front. Cell Infect. Microbiol..

[25] Kang D., Bagchi D., Chen I.A. (2024). Pharmacokinetics and Biodistribution of Phages and their Current Applications in Antimicrobial Therapy. Adv. Ther..

[26] Weber-Dabrowska B., Dabrowski M., Slopek S. (1987). Studies on bacteriophage penetration in patients subjected to phage therapy. Arch. Immunol. Ther. Exp..

[27] Jamalludeen N., Johnson R.P., Shewen P.E., Gyles C.L. (2009). Evaluation of bacteriophages for prevention and treatment of diarrhea due to experimental enterotoxigenic *Escherichia coli* O149 infection of pigs. Vet. Microbiol..

[28] Echterhof A., Dharmaraj T., Blankenberg P., Targ B., Nguyen T.D., Bollyky P.L., Smith N.M., Blankenberg F.G. (2026). Whole-body distribution of three Pseudomonas phages characterized by a translational physiologically based pharmacokinetic model. Antimicrob. Agents Chemother..

[29] Tiwari B.R., Kim S., Rahman M., Kim J. (2011). Antibacterial efficacy of lytic *Pseudomonas* bacteriophage in normal and neutropenic mice models. J. Microbiol..

[30] Dąbrowska K. (2019). Phage therapy: What factors shape phage pharmacokinetics and bioavailability? Systematic and critical review. Med. Res. Rev..

[31] Chibani-Chennoufi S., Sidoti J., Bruttin A., Kutter E., Sarker S., Brüssow H. (2004). In vitro and in vivo bacteriolytic activities of *Escherichia coli* phages: Implications for phage therapy. Antimicrob. Agents Chemother..

[32] Lu J., Wu H., Wu S., Wang S., Fan H., Ruan H., Qiao J., Caiyin Q., Wen M. (2025). Salmonella: Infection mechanism and control strategies. Microbiol. Res..

[33] Wang X., Li J., Ge Z., Fan J., Ma D., Cao H., Shen J., Wang Y., Liu Z., Gomaa S.E. (2025). Phage therapy for intestinal infections: Efficacy, challenges, and future directions in translational research. Front. Microbiol..

[34] Javaudin F., Latour C., Debarbieux L., Lamy-Besnier Q. (2021). Intestinal Bacteriophage Therapy: Looking for Optimal Efficacy. Clin. Microbiol. Rev..

[35] Sarker S.A., Sultana S., Reuteler G., Moine D., Descombes P., Charton F., Bourdin G., McCallin S., Ngom-Bru C., Neville T. (2016). Oral Phage Therapy of Acute Bacterial Diarrhea with Two Coliphage Preparations: A Randomized Trial in Children from Bangladesh. EBioMedicine.

[36] Sulakvelidze A., Alavidze Z., Morris J.G. (2001). Bacteriophage therapy. Antimicrob. Agents Chemother..

[37] Międzybrodzki R., Borysowski J., Weber-Dąbrowska B., Fortuna W., Letkiewicz S., Szufnarowski K., Pawełczyk Z., Rogóż P., Kłak M., Wojtasik E. (2012). Clinical aspects of phage therapy. Adv. Virus Res..

[38] Luong T., Salabarria A.C., Roach D.R. (2020). Phage Therapy in the Resistance Era: Where Do We Stand and Where Are We Going?. Clin. Ther..

[39] Youssef R.A., Sakr M.M., Shebl R.I., Aboshanab K.M. (2025). Recent insights on challenges encountered with phage therapy against gastrointestinal-associated infections. Gut Pathog..

